# Spread of Canine Influenza A(H3N2) Virus, United States

**DOI:** 10.3201/eid2312.170246

**Published:** 2017-12

**Authors:** Ian E.H. Voorhees, Amy L. Glaser, Kathy Toohey-Kurth, Sandra Newbury, Benjamin D. Dalziel, Edward J. Dubovi, Keith Poulsen, Christian Leutenegger, Katriina J.E. Willgert, Laura Brisbane-Cohen, Jill Richardson-Lopez, Edward C. Holmes, Colin R. Parrish

**Affiliations:** Cornell University, Ithaca, New York, USA (I.E.H. Voorhees, A.L. Glaser, E.J. Dubovi, K.J.E. Willgert, L. Brisbane-Cohen, C.R. Parrish);; University of Wisconsin, Madison, Wisconsin, USA (K. Toohey-Kurth, S. Newbury, K. Poulsen);; Oregon State University, Corvallis, Oregon, USA (B.D. Dalziel);; IDEXX Laboratories, West Sacramento, California, USA (C. Leutenegger);; Royal Veterinary College, London, UK (K.J.E. Willgert);; Merck Animal Health, Madison, New Jersey, USA (J. Richardson-Lopez);; University of Sydney, Sydney, New South Wales, Australia (E.C. Holmes)

**Keywords:** canine influenza virus, CIV, H3N2, influenza, dog influenza, United States, South Korea, Chicago, Illinois, viruses, influenza virus, outbreak, zoonoses

## Abstract

A canine influenza A(H3N2) virus emerged in the United States in February–March 2015, causing respiratory disease in dogs. The virus had previously been circulating among dogs in Asia, where it originated through the transfer of an avian-origin influenza virus around 2005 and continues to circulate. Sequence analysis suggests the US outbreak was initiated by a single introduction, in Chicago, of an H3N2 canine influenza virus circulating among dogs in South Korea in 2015. Despite local control measures, the virus has continued circulating among dogs in and around Chicago and has spread to several other areas of the country, particularly Georgia and North Carolina, although these secondary outbreaks appear to have ended within a few months. Some genetic variation has accumulated among the US viruses, with the appearance of regional-temporal lineages. The potential for interspecies transmission and zoonotic events involving this newly emerged influenza A virus is currently unknown.

Influenza A viruses (IAVs) periodically spill over to cause single infections or outbreaks in new host animals. In many cases, these events begin with the transfer of a virus from an avian reservoir host to mammals or domestic poultry, whereas other events result from the transfer of a virus infecting mammals into a new mammalian host. Here we describe the epidemic of an avian-origin canine influenza A(H3N2) virus (H3N2 CIV) in the United States that began by late February 2015 with an outbreak of respiratory disease in dogs in Chicago, Illinois, and nearby areas. Since this time, the virus has circulated continuously among dogs in these areas and has caused sporadic outbreaks nationwide.

H3N2 CIV belongs to the the family *Orthomyxoviridae,* genus *Influenza virus A.* Currently, 18 hemagglutinin (HA) and 11 neuraminidase (NA) subtypes are known to exist. In addition to their reservoir hosts among waterfowl and seabirds, IAVs have infected several other animals in nature to cause epidemics of disease, including repeated outbreaks among humans, swine, horses, terrestrial and domesticated birds, marine mammals, and, more recently, cats and dogs (*1*,*2*). Dogs were not thought to sustain natural IAV infections before the recognition of H3N8 CIV in 2004.

The H3N8 CIV subtype was first detected in 2004 among racing greyhounds in Florida (*3*) and was later shown by serologic testing to have emerged in dogs around 1999 (*4*) through the transfer of an H3N8 equine influenza virus from the Florida clade 1 sublineage of equine influenza virus (*5*). Despite maintaining a relatively low basic reproductive number (R_0_) of ≈1.0 in the general dog population, H3N8 CIV caused outbreaks in many regions of the United States soon after it emerged (*6*). Interconnected networks of dense susceptible host populations found in dog shelters and kennels probably enabled the long-term maintenance of the virus (*6*,*7*). In recent years, however, this virus has been confined to a small area in the northeastern United States (*6*). The reasons for the recent limited circulation of the H3N8 CIV have not been defined, but probably include the apparent inability of the virus to evolve increased transmissibility in the general dog population, increased use of vaccinations in dogs in shelters and kennels, and intensification of control measures in shelters where infections are occurring.

The precise time and place of origin of the H3N2 CIV is still not clear. The virus was first reported in South Korea in 2007, although a virus circulating among dogs in China in 2006 was subsequently reported and sequenced (*8*). Although that virus (A/canine/Guangdong/1/2006 [H3N2]) remains the earliest known H3N2 CIV, serologic evidence shows that H3N2 CIVs were present in South Korea by 2005 (*9*). This timeline agrees with that determined by analysis of sequenced H3N2 CIV isolates from Asia, which points to a single common ancestral virus present in dogs during 1999–2006 (95% highest posterior density) (*10*). Although the timing of the initial H3N2 CIV emergence in dogs is well-supported by serologic surveys and sequence data, the events surrounding the initial emergence are largely unknown. In Asia, the virus appears to be most widespread among dogs in kennels and in meat dog farms and markets (*11*,*12*). Given that live poultry markets in Asia have been identified as a major source of IAVs that spill over to infect new hosts (*13*), close physical contact between birds and dogs in these host-dense environments might have facilitated the emergence of the virus in dogs.

## H3N2 CIV Disease, Host Range, and Zoonotic Potential

Similar to H3N8 CIV infections, H3N2 CIV infections in dogs are associated with mild upper respiratory tract disease, including frequent coughing and fever, although infection of the lungs and more severe disease and death occur on occasion and are probably associated with mixed infections by other viruses or bacteria (*14*). Although CIV epidemics pose a clear threat to canine health, the risks to other animals and humans are largely unknown. Unlike H3N8 CIV, H3N2 CIV appears to have a relatively broad host range, infecting ferrets, guinea pigs, and cats after experimental challenge (*15*,*16*). Nevertheless, experimental inoculation of strains of H3N2 CIV from South Korea and the United States (*17*) into swine resulted in poor replication, suggesting that sustained transmission of the virus after a canine–swine transfer is unlikely, despite swine being a common host of other H3N2 IAVs. Natural spillover of the virus from dogs to cats has been documented in South Korea and the United States, but those outbreaks were largely confined to the shelter populations where they emerged, and the viruses do not appear to undergo prolonged transmission in household cats, despite high levels of viral shedding (*18*,*19*).

To our knowledge, no transfers of either CIV subtype to humans have been documented. However, human pandemic IAVs, including the H1N1 (both seasonal and the 2009 pandemic) (*20*) and the H3N2 (*21*) subtypes, appear able to occasionally infect dogs based on results of serologic testing or isolation of the virus. Although none of these infections is known to have resulted in major onward transmission among dogs, this might provide the opportunity for human IAVs to reassort with CIVs through natural co-infections in dogs. In 2010, a novel H3N1 CIV resulting from the reassortment of an H3N2 CIV (HA segment) and pandemic H1N1/09 virus (the other 7 genomic segments) was isolated in a dog from South Korea (*22*), and in 2012 an H3N2 carrying only the pandemic H1N1/09 matrix segment was isolated from a dog in South Korea (*23*,*24*). In 2015, a novel reassortant H3N2 CIV containing the polymerase acidic (PA) genomic segment from an H9N2 pandemic avian IAV was also isolated from a dog in South Korea (*25*). Additionally, dogs and humans express a similar diversity of sialic acid variants and linkages, which have been demonstrated to be important determinants of IAV infection and host range (*26*), including N-acetyl neuraminic acid and both the α2–3 and α2–6 linkages (*27**,**28*). Given these key biologic and physiologic features and the close contact that exists between human and dog populations, the potential for dogs to act as virus “mixing vessels” or as sources of zoonotic infections by IAVs should not be overlooked.

## The US H3N2 CIV Outbreak

The H3N2 CIV outbreak likely started in February 2015 and spread rapidly through dog training classes, animal shelters, boarding kennels, and veterinary clinics in the Chicago area by early March, at which time initial reports of an unusual respiratory disease in dogs were received (K. Toohey-Kurth and S. Newbury, pers. comm.). We identified clinical samples from infected dogs as IAV-positive in mid-March 2015 by using a type A influenza–specific PCR for the conserved viral matrix segment sequences (*29*) and an amplification protocol approved for use by the National Animal Health Laboratories (Ames, IA, USA). We identified the virus as the H3N2 subtype on April 10, 2015, by using Sanger sequencing of partial HA and matrix genomic segments (performed at Cornell University, Ithaca, NY, USA) and partial NA genomic segments (performed at University of Wisconsin, Madison, WI, USA) that had been amplified as previously described (*29*). Sequences searched in the nucleotide database using blastn (http://blast.ncbi.nlm.nih.gov/Blast.cgi) (*30*) had the highest identity to H3N2 viruses from South Korea. The identification of the virus as the H3N2 subtype and its relation to H3N2 CIV in Asia was announced on April 12 (*31*), and a probable origin in South Korea of the US H3N2 CIV outbreak was confirmed by whole-genome sequencing performed at the National Veterinary Service Laboratories (Ames, IA, USA) on strain A/canine/Illinois/12191/2015, in which all 8 genome segments (GenBank accession nos. KT002533–40) showed highest similarity to H3N2 CIV in South Korea.

**Figure 1:**
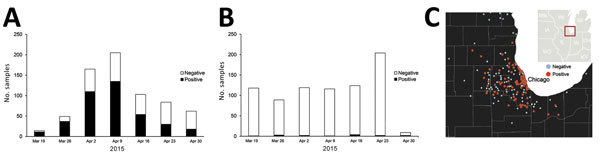
Incidence of canine influenza A(H3N2) virus RNA–positive dogs in the Chicago, Illinois, area, USA, March 14–April 27, 2015. A) Weekly testing summary of samples collected within Illinois. B) Weekly testing summary of samples collected in all other states. C) Presence of virus in the Midwest region, by US postal code.

**Figure 2:**
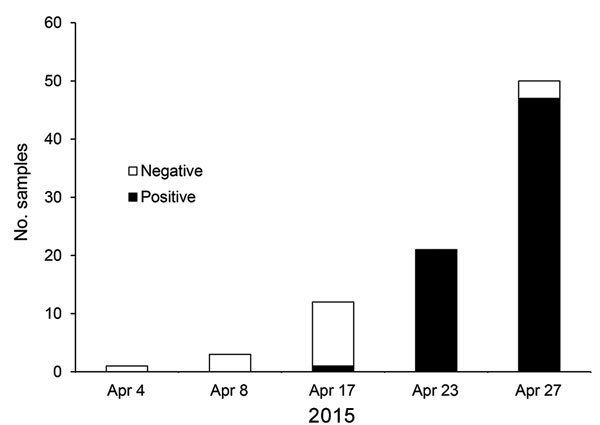
Spread of canine influenza A(H3N2) virus in an animal shelter in the Chicago, Illinois, area, USA, April 2015. The first virus-positive result was obtained on April 17; by April 23, the virus had infected all dogs tested.

During the last weeks of March and into April 2015, H3N2 CIV was detected in many animal shelters in Chicago and in the neighboring areas of Illinois, Indiana, and Wisconsin but was absent from other regions of the United States (Figure 1). The virus spread through affected regions with a wavelike introduction and with case numbers growing rapidly over periods of 2–4 days after introduction into new dog populations (Figures 1, 2). The infection presented as a mild to moderate respiratory disease, often with a characteristic honking cough, with some progression to pneumonia but, generally, with few or no deaths. Some dogs, particularly those in animal shelters, were coinfected with other respiratory pathogens, including canine pneumovirus, canine parainfluenza virus, and canine respiratory coronavirus (*32*). Analyzing the viral loads in shelter dogs during the outbreak showed that the virus peaked in the swabs 2–3 days after the probable time of infection and that in some cases low levels of RNA along with low levels of infectious virus could be detected 2–3 weeks later (*33*) (Table 1), suggesting that prolonged isolation of infected dogs would be necessary to completely prevent transmission. The highest RNA levels detected by real-time reverse transcription PCR were found in nasal swab specimens collected 2–4 days after infection, with lower levels found in other tissues (Table 2) (*32*).

**Table 1:** Persistence of virus or viral RNA in dogs in 2 separate Chicago-area animal shelters that were infected with canine influenza A(H3N2) virus, United States, April 2015*

Shelter and no. days	C_t_ value, by dog no.									
	1	2	3	4	5	6	7	8	9	10
Shelter 1										
1	20.8†	34.7	Neg	29.1	Neg	16.7†	19.0†	34.5	25.2†	25.1†
13	39.3	38.1	31.3	35.1	33.9	31.4	31.9	Neg	29.6	37.3
14	Neg	Neg	32.1	Neg	32	35.3	35.4	38.1	32.3	Neg
15	Neg	Neg	36.2	37.1†	Neg	39.1	Neg	Neg	38.5	Neg
17	Neg	Neg	37.2	Neg	Neg	31.1	Neg	Neg	Neg	Neg
19	Neg	NT	Neg	Neg	Neg	Neg	Neg	Neg	Neg	Neg
20	Neg	NT	Neg	NT	Neg	Neg	Neg	38.2	37.9	37.8†
22	Neg	NT	Neg	NT	Neg	Neg	36.5	39.2	Neg	Neg
24	Neg	NT	Neg	NT	Neg	39.3	Neg	Neg	Neg	37.3
29	Neg	NT	Neg	NT	Neg	Neg	Neg	Neg	Neg	Neg
31	Neg	NT	Neg	NT	Neg	Neg	Neg	Neg	Neg	Neg

	1	2	3	4	5	16
Shelter 2						
1	Neg	37.4	25.1	37.9	26	29.5†
5	NT	NT	22.7	24.6†	NT	NT
6	24.6†	23.7†	NT	NT	NT	NT
10	36.2	39.5	NT	NT	NT	NT
11	NT	NT	NT	NT	37.1	NT
15	NT	NT	38.6†	35.9	38.2†	36
19	NT	NT	Neg	NT	NT	NT
20	37.3	37.2	NT	NT	NT	NT
23	NT	NT	Neg	NT	NT	Neg
24	Neg	Neg	NT	NT	NT	NT
28	Neg	Neg	NT	NT	NT	NT

*Data from Newbury et al. (*33*) Table 1, used with permission. Data shown as rRT-PCR C_t_ values. Values <36 are considered positive results (black cells), and values >37 but <40 are considered weak positive results (dark gray cells). Light gray cells indicate negative rRT-PCR assay results for influenza A virus shedding. C_t_, cycle threshold; Neg, negative; NT, not tested; rRT-PCR, real-time reverse transcription PCR. †Samples for which virus was successfully isolated.

**Table 2:** Results of the rRT-PCR analysis of a necropsied dog in a Chicago-area animal shelter that died after being infected with canine influenza A(H3N2) virus, United State, February 2015*

Type of specimen	C_t_ value
Nasal swab	20.0
Oral swab	34.3
Tracheal swab	36.4
Bronchial swab	28.1
Cranial lung	31.8
Tracheobronchial lymph node	39.6
Liver	Neg
Pancreas	Neg
Ileum	Neg
Kidney	Neg
*Data from Watson et al. (*32*), used with permission. Data shown as rRT-PCR C_t_ values. Values <36 are considered positive results (black cells), and values >37 but <40 are considered weak positive results (dark gray cells). Light gray cells indicate negative rRT-PCR assay results for influenza A virus shedding. C_t_, cycle threshold; Neg, negative; rRT-PCR, real-time reverse transcription PCR.	

Within a few months after its introduction into the Chicago-area dog population, the disease ended in some shelters or kennels, probably because all the resident dogs had become immune. This hypothesis is supported by intensive sampling of the dogs in some shelters, which showed that once the virus entered a closed population of susceptible dogs, most or all dogs would be infected within a few days (Figure 2), thereafter producing a high level of immunologic resistance in that population.

**Figure 3:**
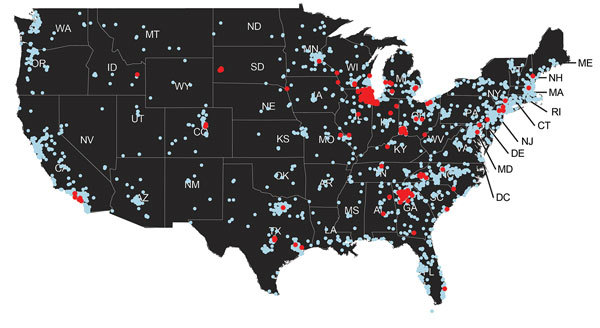
Distribution of clinical samples testing positive (red dots) and negative (blue dots) for canine influenza A(H3N2) virus RNA, United States, March–December 2015.

By May 2015, H3N2 CIV infections were being detected outside of the midwestern United States, and within a year of its introduction, the virus caused substantial outbreaks of disease in several eastern and southeastern states (Table 3, Figure 3). Smaller outbreaks also occurred in Colorado, California, and Washington State, indicating that national spread of the virus occurred. However, none of these secondary outbreaks was sustained or widespread.

**Table 3:** Results of rRT-PCR tests for canine influenza A(H3N2) virus RNA in specimens collected from dogs, by state, United States, February 2015–March 2016*

State/district	Test result	
	Positive	Negative
Alabama	16	355
Alaska	0	22
Arizona	0	223
Arkansas	0	21
California	11	1,601
Colorado	4	137
Connecticut	0	136
Delaware	0	17
District of Columbia	0	5
Florida	1	432
Georgia	559	1,143
Hawaii	0	27
Idaho	1	80
Illinois	820	1,055
Indiana	14	108
Iowa	1	75
Kansas	0	60
Kentucky	5	51
Louisiana	0	132
Maine	1	132
Maryland	3	133
Massachusetts	2	191
Michigan	6	333
Minnesota	5	302
Mississippi	0	39
Missouri	1	115
Montana	2	20
Nebraska	0	27
Nevada	0	39
New Hampshire	0	49
New Jersey	28	175
New Mexico	0	26
New York	3	667
North Carolina	33	475
North Dakota	0	6
Ohio	87	425
Oklahoma	0	18
Oregon	0	84
Pennsylvania	28	1,050
Rhode Island	0	37
South Carolina	2	137
South Dakota	7	14
Tennessee	1	115
Texas	13	833
Utah	0	121
Vermont	0	45
Virginia	0	232
Washington	2	181
West Virginia	1	16
Wisconsin	15	498
Wyoming	0	13
International or unknown	21	609
Total	1,693	12,837

*rRT-PCR, real-time reverse transcription PCR.

## Viral RNA Extraction, Full-Genome Sequencing, and Sequence Analysis

We extracted viral RNA by using the QIAamp Viral RNA Kit (QIAGEN, Valencia, CA, USA) from original clinical sample material obtained from IDEXX Laboratories (West Sacramento, CA, USA) or from the New York State Animal Health and Diagnostic Laboratory at Cornell University (Ithaca, NY, USA), and we adapted to CIV an IAV multisegment real-time reverse transcription PCR amplification approach (*34*). We used amplified genomes to prepare sequencing libraries, which we sequenced on the Illumina MiSeq platform (Illumina, San Diego, CA, USA) with 150 nt paired-end reads, and assembled reads de novo as described in Mena et al. (*34*). We generated 12 CIV full-genome sequences for this study, all of which were submitted to GenBank and assigned accession numbers (Technical Appendix). We obtained additional H3N2 CIV genomes from the National Center for Biotechnology Information Influenza Virus Resource (http://www.ncbi.nlm.nih.gov/genomes/FLU/Database/nph-select.cgi?go = database).

**Figure 4:**
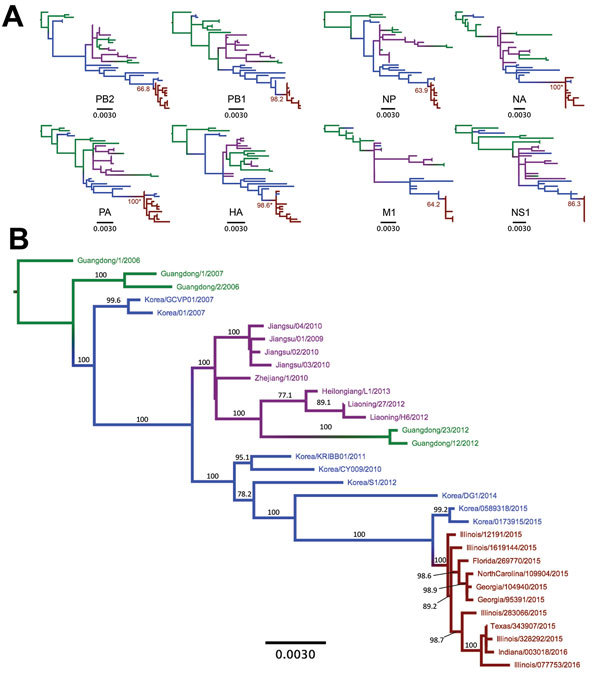
Phylogenetic trees of canine influenza A(H3N2) virus (H3N2 CIV) sequences showing the initial emergence of the virus in southern China (green branches), its appearance in northern and eastern China (magenta branches) and South Korea (blue branches), and its introduction into the United States (red branches). A) Individual genome segment sequences. Red branch numbers indicate bootstrap proportion of US H3N2 CIV clade. Asterisks indicate polyphyletic clades containing US strains and most recent strains from South Korea. B) Concatenated segment phylogenies of all available complete nonreassortant H3N2 CIV genomes. Branch number indicates bootstrap proportions >75. All branch lengths are proportional to the number of nucleotide substitutions per site. All trees rooted by using sequences from the earliest isolated H3N2 CIV. Scale bars indicate nucleotide substitutions per site. HA, hemagglutinin; M1, matrix 1; NA, neuraminidase; NP, nucleocapsid protein; NS1, nonstructural 1; PA, polymerase acidic; PB1, polymerase basic 1; PB2, polymerase basic 2.

We performed consensus sequence editing, alignment, and phylogenetic analyses by using Geneious 9.0.5 and various modules contained in that package (*35*). We trimmed each gene segment to contain only its major open reading frame and aligned the segments by using MAFFT 7.222 (*36*). We then analyzed the segments separately or concatenated with all other genome segment sequences from the same virus. In 39 of the genomes analyzed, we excluded individual segment phylogenies (Figure 4, panel A) and segments from incomplete genomes and intersubtype reassortant viruses. For concatenated full-genome phylogenies (Figure 4, panel B), we excluded intersubtype and intrasubtype reassortant viruses, for a total of 32 genomes analyzed. Total sequence alignment lengths were as follows: polymerase basic 2, 2,277 nt; polymerase basic 1, 2,268–2,271 nt; PA, 2,148 nt; HA, 1,698 nt; nucleocapsid protein, 1,494 nt; NA, 1,407–1,413 nt; matrix 1, 756 nt; and nonstructural 1, 690 nt. Concatenation of the 8 segments yielded a total consensus alignment length of 12,741–12,747 nt. We determined phylogenetic relationships among the sequences by using the maximum-likelihood method available in PhyML (*37*), employing a general time-reversible substitution model, gamma-distributed rate variation among sites, and bootstrap resampling (1,000×). We rooted all trees with the earliest and most basal H3N2 CIV isolate available (A/canine/Guangdong/1/2006 [H3N2]).

## Evolutionary Analysis

As observed previously (*10*), our phylogenetic analysis suggests that H3N2 CIV originated in dogs by the direct transfer of an avian virus in late 2005 or early 2006, with the first virus isolated being in southern China; this virus spread rapidly between different regions of China, to or from South Korea, and to Thailand (no complete full genomes from Thailand are available in the database). Although a single ancestral virus is thought to have given rise to all H3N2 CIVs (*10*), a directly comparable avian virus sequence is not available in the database. However, individual genome segment phylogenies of H3N2 CIV point toward multiple ancestors of the original IAV that gave rise to the canine virus, with lineage sequences from the Americas being observed in the PA segment and lineages from Eurasia being observed in all other segments. Once established in dogs, a handful of hetero- and homo-subtypic reassortment events occurred among the H3N2 CIVs in Asia (*10*). In contrast, our analysis shows that all US H3N2 CIV genome segments exhibit approximately the same tree topology (Figure 4, panel A), suggesting that no large-scale viral reassortment has occurred since its introduction, a conclusion also supported by analysis using the Recombination Detection Program (*38*) (data not shown). Phylogenetic analysis of the concatenated full genome sequences of viruses from the US epidemic show that a single virus from South Korea was introduced into the Chicago area and that the descendants of that virus continue to circulate in that area and have been dispersed widely across the United States (Figures 1,2). The PA, HA, and NA segment phylogenies did not distinguish US viruses from those most recently isolated from South Korea (Figure 4, panel A), confirming the close relationship to the viruses from South Korea and also suggesting that the viral transfer occurred shortly before the virus was recognized in the United States. Despite the short timescale of H3N2 CIV evolution in the United States, some geographic structuring might be present in the data, as indicated by distinct and statistically supported clades (bootstrap proportion >98). For example, a 2015 clade consisting of viruses from Florida, North Carolina, and Georgia probably represents a single introduction of virus to the southeastern United States and a subsequent regional outbreak (Figure 4, panel B).

The US H3N2 CIV and closely related viruses in South Korea show some changes in the sequence adjacent to the receptor binding and antigenic sites of the HA segment. Most notably, a single Gly146Ser amino acid substitution in the HA globular head antigenic site is present in all the US H3N2 CIVs and the most recent CIV in Korea. Because the United States contains a large and naive new host population, with low levels of H3N8 and H3N2 CIV infection or vaccination in most places, it is probably not under selection from antibody immunity during this initial disease emergence, except perhaps among kennel and shelter dogs in the Chicago area.

The exact route of introduction of H3N2 CIV into the United States is unknown. However, whereas an infected dog might shed virus for up to 3 weeks, virus probably remains infectious on fomites for only 12–48 hours. Thus, similar to other IAVs, close host–host contact or direct aerosol exchange is probably the most effective and common route of H3N2 CIV transmission. This hypothesis would suggest that the virus was brought to the United States by infected dogs. Such dogs might have arrived in the United States after being rescued from live animal markets or meat dog farms in South Korea, where the reported overall seroprevalence of H3N2 CIV is ≈19%, with individual dog farms having seroprevalences of up to 100% (*11*). Hundreds of dogs rescued from meat markets in South Korea have been rehomed in the United States since the beginning of 2015 (*39*), although no direct link between any of these dogs and the appearance of H3N2 CIV in the United States has been established.

Likewise, the spread of H3N2 CIV within the United States presumably resulted from movement of infected dogs, rather than from transported fomites, probably through the networks involved in rescuing and rehousing dogs, which would connect the host populations in US dog kennels and shelters. These epidemiologic characteristics resemble those of H3N8 CIV and suggest that control, prevention, and even eradication of the virus in dogs is feasible by controlling the transfer of dogs from infected areas. In addition, with effective inactivated vaccines currently available (*40*) and the possibility of a live-attenuated vaccine (*41*), targeted vaccination of dog populations at high risk will aid in the control of the US H3N2 CIV epidemic. Further monitoring, epidemiologic analysis, and evolutionary studies of H3N2 CIV in the United States will help determine whether these viruses pose a threat to human health and will answer basic questions regarding how IAVs invade and infect new hosts.

Technical Appendix GenBank accession numbers for canine influenza A(H3N2) virus genome segments of strains sequenced.
