# Supplementary material for: Spread of Canine Influenza A(H3N2) Virus, United States
Source: Emerg Infect Dis. 2017 Dec;23(12):1950–7. doi: 10.3201/eid2312.170246 (PMC5708240; doi:10.3201/eid2312.170246)
Supplement: Technical Appendix — GenBank accession numbers for canine influenza A(H3N2) virus genome segments of strains sequenced. [file 17-0246-Techapp-s1.pdf]

Article DOI: https://doi.org/10.3201/eid2312.170246

Spread of Canine Influenza A(H3N2) Virus, United States

Technical Appendix

Technical Appendix Table. GenBank accession numbers for canine influenza A(H3N2) virus genome segments of strains sequenced*

Strain name	PB2	PB1	PA	HA	NP	NA	M	NS
A/canine/Florida/269770/2015(H3N2)	MF173191	MF173194	MF173220	MF173382	MF173216	MF173401	MF173281	MF173142
A/canine/Georgia/104940/2015(H3N2)	KX571013.1	KX570997.1	KX570982.1	KX571026.1	KX571049.1	KX570966.1	KX571051.1	KX570977.1
A/canine/Georgia/95391/2015(H3N2)	KX570974.1	KX570969.1	KX571037.1	KX571027.1	KX571004.1	KX570998.1	KX570980.1	KX570991.1
A/canine/Illinois/077753-16/2016(H3N2)	MF173392	MF173218	MF173226	MF173231	MF173345	MF173298	MF173151	MF173184
A/canine/Illinois/1619144/2015(H3N2)	KX570978.1	KX571041.1	KX570990.1	KX570967.1	KX571038.1	KX570973.1	KX571055.1	KX571044.1
A/canine/Illinois/283066/2015(H3N2)	MF173153	MF173301	MF173398	MF173315	MF173404	MF173329	MF173330	MF173274
A/canine/Illinois/328292/2015(H3N2)	MF173374	MF173173	MF173183	MF173305	MF173310	MF173314	MF173270	MF173225
A/canine/Indiana/003018/2016(H3N2)	MF173221	MF173166	MF173340	MF173371	MF173158	MF173113	MF173227	MF173288
A/canine/Korea/0173915/2015(H3N2)	MF173248	MF173109	MF173277	MF173138	MF173293	MF173171	MF173346	MF173349
A/canine/Korea/0589318/2015(H3N2)	KX571005.1	KX571025.1	KX571012.1	KX571053.1	KX570994.1	KX571019.1	KX571045.1	KX570993.1
A/canine/North Carolina/109904/2015(H3N2)	KX570975.2	KX571003.2	KX570965.2	KX571008.2	KX570964.2	KX571007.2	KX571020.2	KX570971.2
A/canine/Texas/343907/2015(H3N2)	KX571029.2	KX571032.1	KX571018.2	KX571042.2	KX571021.2	KX571043.2	KX571054.1	KX571033.2

*Accession numbers beginning with MF are pending release as of publication and therefore lack the 1 suffix. HA, hemagglutinin; M1, matrix 1; NA, neuraminidase; NP, nucleocapsid protein; NS1, nonstructural 1; PA, polymerase acidic; PB1, polymerase basic 1; PB2, polymerase basic 2.
